# A Case of IFAP Syndrome with Severe Atopic Dermatitis

**DOI:** 10.1155/2015/450937

**Published:** 2015-01-21

**Authors:** Catarina Araújo, Miguel Gonçalves-Rocha, Cristina Resende, Ana Paula Vieira, Celeste Brito

**Affiliations:** ^1^Dermatology Department, Hospital de Braga, Sete Fontes, São Victor, 4710-243 Braga, Portugal; ^2^Medical Genetics Unit, Hospital de Braga, Sete Fontes, São Victor, 4710-243 Braga, Portugal

## Abstract

*Introduction.* The IFAP syndrome is a rare X-linked genetic disorder characterized by the triad of follicular ichthyosis, atrichia, and photophobia. *Case Report.* A three-month-old Caucasian, male patient was observed with noncicatricial universal alopecia and persistent eczema from birth. He had dystrophic nails, spiky follicular hyperkeratosis, and photophobia which became apparent at the first year of life. Short stature and psychomotor developmental delay were also noticed. Histopathological examination of skin biopsy on left thigh showed epidermis with irregular acanthosis, lamellar orthokeratotic hyperkeratosis, and hair follicles fulfilled by parakeratotic hyperkeratosis. The chromosomal study showed a karyotype 46, XY. Total IgE was 374 IU/mL. One missense mutation c.1360G>C (p.Ala454Pro) in hemizygosity was detected on the *MBTPS2* gene thus confirming the diagnosis of IFAP syndrome. *Conclusions.* We describe a boy with a typical clinical presentation of IFAP syndrome and severe atopic manifestations. A novel missense mutation c.1360G>C (p.Ala454Pro) in *MBTPS2* gene was observed. The phenotypic expression of disease is quantitatively related to a reduced function of a key cellular regulatory system affecting cholesterol and endoplasmic reticulum homeostasis. It can cause epithelial disturbance with failure in differentiation of epidermal structures and abnormal skin permeability barrier. However, no correlation phenotype/genotype could be established.

## 1. Introduction

The ichthyosis follicularis, atrichia, and photophobia (IFAP) syndrome is a rare genodermatosis first described by MacLeod in 1909 [1]. Up to forty cases were described and most of these had additional features including atopic eczema in 36% cases [2].

The X-linked recessive mode of inheritance was the first reported and the gene mapped to the 5.4 Mb region between DXS989 and DXS8019 on Xp22.11-p22.13. Later an autosomal pattern has been considered [3]. More recently the missense mutation of the* MBTPS2* gene (membrane-bound transcription factor protease site 2), which codes for an intramembrane zinc metalloprotease, was found to be essential for cholesterol homeostasis and endoplasmic reticulum stress response [2].

All affected males have the IFAP triad of follicular ichthyosis, atrichia of the scalp, and photophobia of varying degree from birth. Affected or carrier females may display some of its clinical features such as asymmetric distribution of body hair, patchy alopecia, and linear lesions of follicular ichthyosis that follows the lines of Blaschko [1].

## 2. Case Report

A three-month-old white male patient was referred to the dermatology department with universal alopecia. The boy was born underweight (1570 gr) and preterm at 35 weeks and 5 days by natural birth. Pregnancy was uneventful. He has nonconsanguineous Caucasian parents and there was no history of similar illness in the family or familiar history of atopy.

The dermatological examination showed skin phototype II with a complete absence of scalp hair, eyebrows, and eyelashes. He had a prominent forehead, large ears, generalized dryness of the skin, and follicular papules and pustules on an erythematous base scattered on the trunk and upper and lower limbs. A skin biopsy from left arm showed inflammatory neutrophilic infiltrate involving adventitial dermis and a hair follicle with small follicular abscess. A survey of infectious agents was negative.

One month later, parents reported severe itching and persistent and exudative eczema of the folds mainly in the posterior aspect of the neck and in the groin was observed; cheilitis and blepharitis were also noted. He underwent conservative treatment for atopic dermatitis with moisturizers and topical steroids.

By 4 months of age, he developed a bilateral inguinal hernia which required surgical correction.

**Figure 1:**
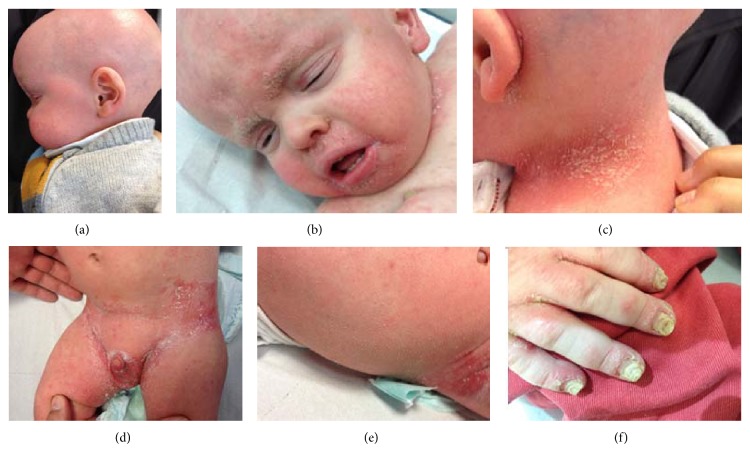
Clinical appearance and dermatologic findings of the patient. Note the alopecia with an absence of scalp hair, eyebrows, and lashes (a). Spiky follicular hyperkeratosis on the trunk. Skin was dry and scaly and erythematous follicular papules were observed over posterior aspect of the neck and groin ((c), (d), and (e)), cheilitis (b), and dystrophic nails (f).

The dermatological examination at 6 months revealed dystrophic nails and spiky follicular hyperkeratosis for the extensor surfaces and scalp symmetrically distributed. The patient kept widespread severe itching and exudative eczema mainly in the posterior aspect of the neck and groin with great interference in sleep (Figure 1).

Investigation, including complete blood count, liver function tests, serum lipids, immunoglobulins, T-cell subsets, and leukocyte function studies, was normal. Amino acid studies of plasma and urine, thyroid function, zinc, folic acid, ferritin, and urinalysis were unremarkable. Lymphocyte chromosome study revealed a 46, XY karyotype. Total IgE was 374 IU/mL.

**Figure 2:**
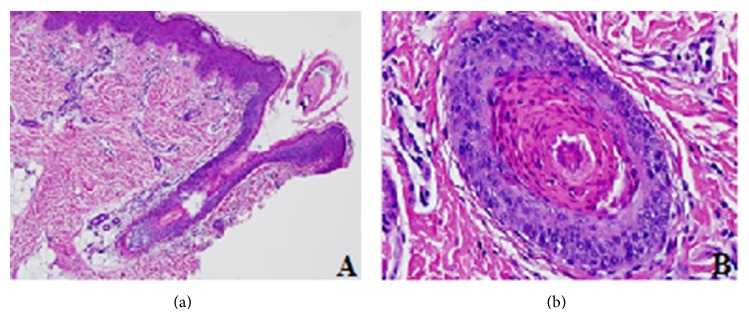
A skin biopsy from left thigh performed at 12 months showed epidermis with irregular acanthosis and orthokeratotic lamellar hyperkeratosis (a). The hair follicles demonstrate absence of hair shaft and are fulfilled by parakeratotic hyperkeratosis (b).

At this point he was observed by a Medical Genetics consultant where the IFAP syndrome was suspected and a new skin biopsy from an area without atopic lesions was requested. A new skin biopsy from left thigh performed at 12 months showed epidermis with irregular acanthosis and orthokeratotic lamellar hyperkeratosis (Figure 2). The hair follicles revealed absence of hair shaft which were fulfilled by parakeratotic hyperkeratosis. The sweat glands were normal.

Photophobia became apparent within first years of life. He was able to walk without help at around 21 months. He had global developmental delay and, till the age of 28 months, there was no speech acquisition though understanding simple commands. Dental development was normal. Sweating was not found to be impaired.

Slit lamp examination revealed the presence of a slight punctate epithelial keratopathy. Visual acuity was not determined. Neurological examination and auditory brainstem response were normal. The left renal pelvis was more prominent. Abdominal ultrasound and echocardiogram were unremarkable.

At the age of 16 months, sequencing of* MBTPS2* gene revealed a missense mutation c.1360G>C (p.Ala454Pro) in hemizygosity, thus validating the diagnosis of ichthyosis follicularis with alopecia and photophobia (IFAP-OMIM number 308205).

The patient remains in followup at dermatology with inflammatory skin disease characterized by relapses and remissions of intensely pruritic lesions with a significant impact on quality of life of the patient and his family. He is treated for relapses with cycles of betamethasone oral solution 0.5 mg/mL.

## 3. Discussion

We describe a boy with a typical clinical presentation of IFAP syndrome and severe atopic manifestations.

Dermatologic findings are variable. Congenital alopecia is a distinct clinical finding, which typically involves the scalp, eyebrows, and eyelashes. There is also complete body hair alopecia and, in some cases, thin and sparse hair [1]. The skin of the infant is characterized by widespread noninflammatory thorn-like follicular projections and symmetrically hyperkeratotic spiny papules with a predilection for the extensor surfaces and scalp [4]. Histologically, there are keratinous follicular plugging and parakeratosis. There is a thickened granular layer at the infundibulum with a normal perifollicular granular layer. Poorly developed hair follicles, tending to be surrounded by an inflammatory infiltrate, reduced eccrine sweat glands, and absence of sebaceous glands are typical findings [5]. Other cutaneous manifestations include psoriasiform plaques, angular cheilitis, periungual inflammation, hypohidrosis, and atopic eczema. Dystrophic nails are reported. Dental development is normal.

Ocular [1, 6–8], neurological [6, 9], and other clinical manifestations associated with IFAP syndrome [10, 11] are described in Table 1. Photophobia may exist since birth or may develop later in childhood [1, 7]. It is postulated that a defect in Bowman membrane results in superficial corneal ulceration and progressive corneal scarring [8]. Vision acuity is generally low [8]. Global developmental delay is a common feature in most patients.

Generalized ichthyosis and alopecia have been reported in very few syndromes. In our patient, consideration was given to three diagnoses: ectodermal dysplasia, keratitis-ichthyosis-deafness syndrome (KID), and the IFAP syndrome.

Ectodermal dysplasia is a group of syndromes affecting the development or function of the ectodermal structures. It is characterized by sparse hair, abnormal or missing teeth, and inability to sweat [12]. Ectodermal dysplasia can also be seen in IFAP syndrome as additional feature [13].

KID syndrome shares many features with IFAP although most people with KID syndrome have thick, hard skin on the palms of the hands and soles of the feet (palmoplantar keratoderma) and hyperkeratotic plaques with an erythematous base (erythrokeratoderma rather than ichthyosis). Nevertheless, in patients with KID teeth may be malformed and ocular changes (keratitis and photophobia) are usually observed during the second and third decades of life [14]. In addition, congenital sensorineural hearing loss in this condition is usually important and the mode of inheritance is autosomal dominant.

Clinical manifestations identified in our patient were consistent with the IFAP syndrome but with severe atopic skin lesions less common in other patients [15, 16]. The diagnosis was made although the variable phenotypes which share similar clinical features with other genodermatoses and this case report outline the variability of the clinical spectrum.

The diagnosis is based on the clinical features and the presence of missense mutations exchanging highly conserved amino acids of membrane-bound transcription factor protease site 2. This is a membrane-embedded zinc metalloprotease that activates signaling proteins involved in sterol control of transcription and endoplasmic reticulum (ER) stress response [10]. Skin histopathology is nonspecific. One missense mutation c.1360G>C (p.Ala454Pro) in hemizygosity was detected. This variant observed in* MBTPS2* is a novel mutation not yet described. If association of IFAP syndrome and severe atopic dermatitis may be fortuitous, the phenotypic expression of disease is quantitatively related to a reduced function of a key cellular regulatory system affecting cholesterol or ER homeostasis. It can cause epithelial disturbance with failure in differentiation of epidermal structures and abnormal skin permeability barrier. However, no correlation phenotype/genotype could be established.

Given the complex pathophysiology of atopic dermatitis, the therapeutic goal is essentially a multipronged approach that aimed to repair and protect the skin barrier, reduce microbial colonization and immune regulation to greater therapeutic efficacy, and improve quality of life of these patients. Routine symptomatic treatment of the generalized follicular hyperkeratosis is performed with topical keratolytics and emollients. Oral retinoids have previously been used with moderate response in cutaneous features in other boys on oral acitretin at a dosage of 0.3 to 1 mg/kg/day. However, photophobia and alopecia did not improve [17].

Life expectancy in patients with IFAP syndrome can vary from death in the neonatal period to normal survival. Cardiopulmonary complications remain the major cause of death [14].

IFAP syndrome cannot be detected prenatally by ultrasonography. The mutation might also arise in the patient* de novo* [2]. If the mutation has been previously detected in the family, prenatal diagnosis can be offered to female carriers. The patient's mother was healthy with no relevant medical or surgical history. Currently, genetic study of the mother is ongoing.

## 4. Conclusion

IFAP syndrome is an X-linked genodermatosis with variable severity and genetic mutations that were found to imply* MBTPS2* gene as causal. Modifying factors might modulate the phenotype in this syndrome and a better knowledge of the* MBTPS2* gene may help understand the mechanism of its enzymatic action. The prognosis is mainly determined by cutaneous and extracutaneous manifestations so a multidisciplinary approach to these patients is imperative.

## Figures and Tables

**Table 1:** 

Clinical features in IFAP syndrome	
Ocular	Photophobia, corneal scars, punctate keratopathy, corneal erosion and neovascularization, and atopic keratoconjunctivitis

Neurological	Global developmental delay, seizures, and mild inner cerebral atrophy

Other	Short stature, dysmorphic features such as frontal bossing, choanal atresia, and large ears. Intestinal anomalies such as omphalocele, Hirschsprung's disease, congenital aganglionic megacolon, stenosis of the small intestine, renal, vertebral, and testicular anomalies, inguinal hernia, cleft hands, and recurrent infections
